# Questions about the use of antibiotics in acute pancreatitis

**DOI:** 10.1186/1749-7922-1-20

**Published:** 2006-07-04

**Authors:** Tercio De Campos, Jose Cesar Assef, Samir Rasslan

**Affiliations:** 1 Emergency Surgery Unit, Santa Casa School of Medical Sciences, São Paulo, Brazil

## Abstract

**Background and objective:**

The use of antibiotics in acute pancreatitis despite recent clinical trials remains controversial. The aim of this study is to review the latest clinical trials and guidelines about antibiotics in acute pancreatitis and determine its proper use.

**Methods:**

Through a Medline search, we selected and analyzed pertinent randomized clinical trials and guidelines that evaluated the use of antibiotics in acute pancreatitis. We answered the most frequent questions about this topic.

**Results and conclusion:**

Based on these clinical trials and guidelines, we conclude that the best treatment currently is the use of antibiotics in patients with severe acute pancreatitis with more than 30% of pancreatic necrosis. The best option for the treatment is Imipenem 3 × 500 mg/day i.v. for 14 days. Alternatively, Ciprofloxacin 2 × 400 mg/day i.v. associated with Metronidazole 3 × 500 mg for 14 days can also be considered as an option.

## Review

Severe acute pancreatitis (SAP) as defined by the Atlanta criteria, is present in up to 25% of patients with acute pancreatitis (AP) [1], with mortality of 10%–20%.

In these patients, AP develops in two phases: the first ten days are characterized by the systemic inflammatory response syndrome (SIRS), whereas in the end of the second week infection complications begin to appear [2].

These complications due to infection are responsible for up to 80% of deaths in patients with AP. About 40% to 75% of patients with pancreatic necrosis develop infection, 24% after the first week and 71% after the third week [3,4], with mortality rates up to 50% [5,6]. The mechanisms of infection in AP are bacteria translocation, via lymphatic, via hematogenic, and reflux from duodenum and biliary tree as well [7].

If the major problem in AP is infection of pancreatic necrosis, it makes sense to consider that the use of antibiotics in this situation could reduce the morbidity and mortality of these patients. However, at present there are still controversies about this topic.

Therefore, some questions remain unclear regarding the use of antibiotics in AP:

1-Should we use antibiotics in Acute Pancreatitis?

2-Which antibiotic?

3-How long?

4-Should we wait for more studies?

### 1- Should we use antibiotics in Acute Pancreatitis?

SAP is characterized by the presence of organic failure (Ranson ≥ 3 or APACHE II ≥ 8), or local complications such as necrosis, pseudocyst and abscess [8-11].

The Balthazar score on CT scan determines the intensity of local complications. The presence of pancreatic or peri-pancreatic necrosis and fluid collections define AP as locally severe [12,13].

Some authors observed reduction in necrosis infection after the introduction of antibiotics in the treatment of severe forms of AP, when compared with historic controls without antibiotics. Banks et al. described a decrease from 67% to 32% [14], while Ho & Frey observed a reduction from 75% to 20% in the infection of pancreatic necrosis [15].

#### a. Review of clinical trials

There are few randomized clinical trials about the use of antibiotics in AP. Pederzoli et al. in 1993 [16] conducted the first of them, which analyzed 74 patients with SAP in six medical centers in Italy. The patients were randomized in two groups: one control with 33 patients and the treatment group with 41 patients that received Imipenem 3 × 500 mg/day intravenously (i.v.) during 14 days. They observed reduction in pancreatic sepsis in patients that received antibiotics (30.3% vs. 12.2%, p < 0.01). However, they did not find reduction in mortality between the groups (12% vs. 7%, respectively).

Sainio et al. carried out another study in 1995 [17], analyzing data from 60 patients with SAP. One group control and the other treated with Cefuroxime 3 × 1.5 g/day i.v. They observed reduction in the number of pancreatic operations (36 vs. 8, p = 0.012) and in mortality (7 vs. 1, p = 0,028) in the treatment group. However, 66.7% of patients in this group had their antibiotics changed during the treatment. Furthermore, 23 out of 30 patients in the control group received antibiotics in an average period of six days after the beginning of the treatment. The use of this cephalosporin is also questionable due to low pancreatic penetration.

Delcenserie et al. in 1996 [18] analyzed prospectively 23 patients with alcoholic SAP that were divided into two groups: one control and one that was treated with Ceftazidime 3 × 2 g/day i.v., Amicacin 2 × 7.5 mg/kg i.v., and Metronidazole 3 × 500 mg i.v. for 10 days. They observed a decrease in septic complications in the treatment group (7 vs. 0, p = 0.03). Nevertheless, there was no difference in mortality rate between the groups. In another clinical trial, Schwarz et al. described a decrease in pancreatic infection in a group of 29 patients, utilizing Ofloxacin and Metronidazole (7% × 46%, respectively) [19].

In summary, the studies of Pederzoli, Delcenserie e Schwarz demonstrated reduction in pancreatic infection, although without decrease in mortality. Only Sainio observed reduction in mortality with the use of antibiotics. In spite of these results, a solid answer to the question about the use of antibiotics in AP was still missing [1,20] (Table 1).

**Table 1:** Clinical studies comparing the use of antibiotics with placebo

Authors	Treatment	Patients (n)	Infected necrosis (%)	Mortality (%)
Pederzoli et al. [16]	Imipenem	41	12*	7
	Placebo	33	30	12
Sainio et al. [17]	Cefuroxime	30	30	3*
	Placebo	30	40	23
Delcenserie et al. [18]	Ceftazidime + Amicacin + Metronidazole	11	0*	0
	Placebo	12	33	25
Schwarz et al. [19]	Ofloxacin + Metronidazole	13	62	0
	Placebo	13	54	15

In 2004, the Ulm group in Germany published the best study regarding the use of antibiotics in AP [21]. It was a prospective randomized double-blind trial that analyzed 114 patients, 58 that received antibiotics (Ciprofloxacin 2 × 400 mg/day i.v. + Metronidazole 2 × 500 mg/day i.v.) and 56 that received placebo. It was established that if any patient developed systemic inflammatory response, organic failure, any kind of infection or clinical deterioration, this patient would be discontinued from the protocol with open antibiotic treatment. Their results showed that the use of antibiotics did not reduce pancreatic infection (12% antibiotics vs. 9% placebo, p = n.s.) and mortality (5% antibiotics vs. 7% placebo, p = n.s.). However, 28% of patients in the group that received antibiotics had their protocol opened, versus 46% in the placebo group (p = 0.037). Furthermore, the mean time to open the protocol was 11.5 days in the treatment group and 5 days in the placebo group (Table 2). Based on these data, it is reasonable to suppose that a group of patients had benefits receiving early antibiotics. This is the same opinion of other authors [22,23].

**Table 2:** Results of a clinical double-blind trial about the use of antibiotics in severe acute pancreatitis [21]

	Intention to treat (114 patients)		Pancreatic necrosis (CT) (76 patients)	
Treatment	Ciprofloxacin/Metronidazole (58 patients)	Placebo (56 patients)	Ciprofloxacin/Metronidazole (58 patients)	Placebo (56 patients)
Mortality	5%	7%	7%	11%
Surgical treatment	17%	11%	24%	19%
Necrosis infection	12%	9%	17%	14%
Protocol opened	28%*	46%	37%	57%

#### b. Time of infection

Although the risk for the development of pancreatic infection is higher in the third week, microorganisms can be found in the pancreatic tissue in the first week [24].

De Souza et al. demonstrated in an experimental model that bacteria could be detected in the pancreatic tissue six hours after the induction of AP [25]. Similarly, Schwarz et al. observed bacteria in the pancreatic necrosis between 8 and 16 hours after the pancreatitis induction [26].

These data suggest that the best time to introduce antibiotics is immediately after the diagnosis of AP and the evaluation of its severity. However, the confirmation of necrotizing pancreatitis by CT scan can take at least 72 hours since the onset of symptoms. C-reactive protein is a sensitive marker of pancreatic necrosis and it starts to increase significantly 48 hours after the onset of symptoms. Thus, C-reactive protein can be useful in the identification of patients with high possibility to develop necrosis, in particular when the value is over 150 mg/dl, and subsequently define which patients are candidates to receive early antibiotics [27].

Nordback et al, in a clinical trial observed advantages in the early use of Imipenem in patients with pancreatic necrosis when compared with the late use [28].

#### c. Complications of antibiotics in AP

The main arguments against the use of antibiotics are the increase of fungal infection, and the increase of bacteria resistance, with more Gram-positive infections [1].

There are evidences in the literature that fungal infection has to be considered as an additional factor which influences the outcome of the patients [29].

One paper analyzing data of 46 patients with infected pancreatic necrosis receiving antibiotics showed that 17 (37%) of them developed fungal infection. Nevertheless, the fungal infection did not contribute to increase mortality in this group of patients [30].

Gloor et al. analyzed 103 patients with necrotizing AP that received antibiotics (Imipenem/cilastatin) and concluded that fungal infections when treated properly do not contribute to a worst prognosis. This same paper showed that the presence of multi-resistant bacteria was rare (2.9%) [3].

#### d. Guidelines

Several guidelines about the treatment of AP can be found in the literature, and the majority of them recommend the early use of antibiotics in patients with pancreatic necrosis (Table 3) [31-39]. The International Association of Pancreatology guidelines for the management of AP recommends the use of prophylactic broadspectrum antibiotics to reduce infection rates in CT-proven necrotizing pancreatitis as a recommendation grade A [31]. The Cochrane review about the use of antibiotics in AP concluded that despite variations in drug agent, duration of treatment and methodological quality of clinical trials, there is strong evidence that intravenous antibiotic prophylactic therapy for 10 to 14 days decrease the risk of superinfection of necrotic tissue and mortality in patients with SAP with proven pancreatic necrosis at CT [37].

**Table 3:** Consensus recommendations about the use of antibiotics in acute pancreatitis

Consensus	Origin	Recommendation
Uhl et al. 2002 [31]	International Association of Pancreatology (Pancreatology)	Yes
Toouli et al. 2002 [32]	Journal of Gastroenterology and Hepatology	Yes
Nathens et al. 2004 [33]	Crit Care Med	No
UK working party 2005 [34]	United Kingdom (Gut)	No consensus
Clancy et al. 2005 [35]	Journal of Gastrointestinal Surgery	Yes
Werner et al. 2005 [36]	Gut	Yes
Bassi et al. 2003 [37]	Cochrane	Yes
Chinese pancreatic disease group 2005 [38]	Chinese society of gastroenterology (Chinese Journal of Digestive Diseases)	Yes
Takeda et al. 2006 [39]	JPN Guidelines for the management of acute pancreatitis	Yes

Moreover, the UK guidelines for the management of AP advocate that the risk of infected necrosis is very small when there is less than 30% necrosis [34]. Although there is no support in the literature, it is reasonable to assume that prophylactic antibiotic therapy should be considered only for patients with more than 30% pancreatic necrosis. In contrast, there is no evidence in the literature that the use of antibiotics in the absence of necrosis is beneficial.

Based on these studies we believe that the use of antibiotics is benefic to patients with SAP with more than 30% pancreatic necrosis, and starting as soon as possible.

### 2- Which antibiotic?

Two main aspects must drive the choice of the antibiotics: the flora and the penetration in the pancreatic tissue.

One single microorganism causes most of infections in AP. The bacteria most frequently found are E. coli (27–35%), Enterococcus (24–26%), Staphylococcus aureus (14–16%), Staphylococcus epidermidis (15%), Klebsiella (15%) Pseudomonas sp (7–11%) and Streptococos (4–7%) [7,40,41].

Ampicillin was one of the first antibiotics used in AP, although it has failed to show advantages to these patients, due to poor action in the flora and low penetration in the pancreatic tissue [42].

Experimental studies were performed to determine which antibiotic would have a better pancreatic concentration [43,44]. Mithofer et al. in a necrotizing pancreatitis model defined three groups: one treated with saline solution, the second treated with Ciprofloxacin and the third treated with Imipenem/cilastatin six hours after the induction of AP. After seven days, 75% of the animals in the saline group had infection, while 25% in the Imipenem group (p < 0.01) and 6% in the Ciprofloxacin group (p < 0.01). After 21 days, the infection rate was 71%, 33% and 35% respectively (p = n.s.). The survival in seven days was 68% in the control group, 60% in the Imipenem group (p = 0.071) and 43% in the Ciprofloxacin group (p < 0.01). After 21 days, the survival was 23% in the control group vs. 52% in the Imipenem group (p = 0.044) and 70% in the Ciprofloxacin group (p < 0.001) [44].

Büchler et al, in 1992, developed a table about the efficacy of several antibiotics, based on the pancreatic concentration of the antibiotic in patients undergone pancreatic surgery (Table 4) [45]. The antibiotic with higher penetration was Imipenem, followed by Ciprofloxacin.

**Table 4:** Penetration of antibiotics in the pancreatic tissue [45]

Poor penetration	Medium penetration	Good penetration
Netilmicin	Mezlocillin	Ciprofloxacin
Tobramicin	Piperacillin	Ofloxacin
	Ceftizoxime	Imipenem
	Cefotaxime	Metronidazole

The pancreatic concentration of an antibiotic is determined by some factors: properties of the antibiotic, anatomy and physiology of the pancreas, alkaline pH, high ion concentration, enzymatic and hormonal regulation, pathologic changes and infection [45]. Spicak et al., studied experimentally the penetration of five antibiotics in the pancreatic tissue of rats with AP, and observed that Cefoperazone (3rd generation cephalosporin) and Ofloxacin had adequate penetration, while Amoxicillin/Clavulanic acid, Piperacillin and Amicacin demonstrated insufficient penetration. They concluded that antibiotic penetration was not influenced by necrosis of the pancreas [46].

In a clinical trial, Bassi et al. compared two groups with 30 patients each, whereas the first one received Pefloxacin, and the second Imipenem. They observed a decrease in the infection of pancreatic necrosis in the Imipenem group (3 × 10, p = 0.034). There was no difference in mortality (10% contra 24%, p = 0.18) [47]. This study showed that Pefloxacin was less effective than Imipenem in clinical practice. Although this is not a study comparing Ciproflofloxacin, it can suggest that antibiotics of this class can be less effective than Imipenem.

Based on these studies and guidelines, we conclude that the recommended antibiotic in AP is Imipenem 3 × 500 mg/day i.v. Alternatively, the use of Ciprofloxacin 2 × 400 mg/day i.v. associated with Metronidazole 3 × 500 mg/day i.v. can also be considered.

### 3- How long?

There are no studies evaluating how long these patients should be treated with antibiotics. This time is determined by consensus meetings that recommend a period of 10 to 14 days [32,34,35,37,38]. After this period, each patient must be evaluated individually to determine if the antibiotics will be suspended, modified or continued. The cultures for bacteria and fungus are fundamental in this phase.

### 4- Should we wait for more studies?

Although there is no definitive conclusion about the use of antibiotics in AP, strong evidences suggest that they are beneficial. Isenmann's study is the more adequate about the use of antibiotics, and despite of a negative conclusion, it has shown that a group of patients deserved the early use of antibiotics.

New studies are coming, hopefully with a greater number of patients and more solid conclusions. However, we should not wait for them to make a decision in the treatment of these patients.

## Conclusion

Based on available studies and in the guidelines opinions, we conclude that the best policy currently is the use of antibiotics in patients with SAP and more than 30% pancreatic necrosis. The antibiotic recommended is Imipenem 3 × 500 mg/day i.v. for 14 days. Alternatively, Ciprofloxacin 2 × 400 mg/day i.v. associated with Metronidazole 3 × 500 mg/day for 14 days can also be considered as an option.

## Abbreviations

AP: acute pancreatitis

i.v.: intravenously

SAP: severe acute pancreatitis

SIRS: systemic inflammatory response syndrome

## Competing interests

The author(s) declare that they have no competing interests.

## Authors' contributions

TDC carried out acquisition, analysis, interpretation of the data and drafting of the manuscript.

JCA was involved in the interpretation of the data and drafting of the manuscript.

SR revised critically the manuscript for the intellectual content till the final version.

Authors have read and approved the final manuscript.
